# Understanding the Physiological Requirements of the Mountain Bike Cross-Country Olympic Race Format

**DOI:** 10.3389/fphys.2018.01062

**Published:** 2018-08-09

**Authors:** Arnaud Hays, Simon Devys, Denis Bertin, Laurie-anne Marquet, Jeanick Brisswalter

**Affiliations:** ^1^Institut des Sciences du Mouvement, Aix-Marseille Université, Marseille, France; ^2^Université Côte d’Azur, LAMHESS, Nice, France

**Keywords:** off-road cycling, maximal oxygen uptake, power output, acyclical, performance monitoring, XCO mountain bike, heart rate

## Abstract

**Objectives:** To evaluate the physiological requirements imposed by the current mountain biking Cross-Country Olympic (XCO) format.

**Methods:** Sixteen Cross-Country cyclists competing at national or international level participated in this study. All participants completed a simulated and a real official race on a cycling-accredited race track. Oxygen consumption (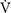
O_2_) and heart rate (HR) values expressed as %
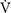
O_2max_ and %HR_max_, respectively, were divided into three physiological intensity zones. The first zone (Z1) was the physiological region below VT1, the second zone (Z2) corresponded to a region between VT1 and VT2, and the third zone (Z3) was located between VT2 and VO_2max_. For power output, an additional fourth zone was considered above maximal aerobic power (MAP).

**Results:** When competing in the current XCO format, 37.0 ± 17.9% of the race is performed above the second ventilatory threshold at a mean intensity of 87% 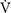
O_2max_ and 25% of the race was spent above MAP. This contribution varied between laps, with a very high intensity during the first lap and more aerobic subsequent laps. The durations of most of the periods beyond MAP oscillated between 5 and 30 s. Between these short, repeated bursts, low-intensity periods of exercise were recorded.

**Conclusion:** The current XCO race format is an acyclical and intermittent exercise comparable to high-intensity team sports. Moreover, our results highlight the relevance of 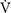
O_2_ values when analyzing XCO performance, they should be combined with commonly used HR and/or power output data.

## Introduction

Since cross-country mountain biking (XCO) was first recognized as an Olympic sport in 1996, the discipline has significantly evolved. In particular, in 2007 the race duration was reduced from 2 h 30 to 1 h 30 while increasing the technical constraints. Current Union Cycliste Internationale (UCI) rules require Olympic cross-country XCO events to feature a lap length of 4–6 km, a race time of around 1 h 30–1 h 45 and a variety of terrains, from forest-style tracks to gravel paths, and include numerous jumps, climbs and descents (UCI Regulations, 2018).

Most previous scientific studies on the discipline were conducted before 2007 (Gregory, 2002; Impellizzeri et al., 2002; Lee et al., 2002; Stapelfeldt et al., 2004; Impellizzeri and Marcora, 2007). Results from several studies indicated that aerobic power (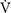
O_2max_) and maximal aerobic power output (MAP) correlated strongly with mountain bike XCO competition performance (*r* = 0.6–0.9) (Impellizzeri, 2005; Gregory et al., 2007; Prins et al., 2007). However, Impellizzeri (2005) reported that only ∼40% of the variance in performance could be explained by these physiological parameters and the remainder was unexplained. More recently, Inoue et al. (2012) examined the correlation between XCO race time and anaerobic power using a Wingate test. Their results suggested that anaerobic power is also an important determinant of performance in competitive XCO (*r* = -0.79). Furthermore, for complex intermittent sports like mountain biking, the physiological demand of the race is difficult to assess in laboratory conditions (Reilly et al., 2009). Field-based methods are therefore better suited to assess the variability of the demands of XCO form, which is characterized by an explosive rhythm at the start of the race, followed by intermittent bursts (Stapelfeldt et al., 2004), high variability in power, direction, speed and cadence in the different portions of each lap and the role played by upper body muscles in resisting vibrations during downhill sections (Hurst and Atkins, 2006).

In most studies examining simulated races performed in an ecological context, XCO characteristics were described using smoothed heart rate (HR) and power output data (Stapelfeldt et al., 2004; Gregory et al., 2007), with little regard for the intermittent nature of the mechanical workload required for different parts of the race. More recently, Viana et al. (2018) and Granier et al. (2018) examined pacing strategies during the different laps of the current XCO format during either simulated (Viana et al., 2018) or real races (Granier et al., 2018). Their results indicated that all cyclists adopt similar pacing strategies between laps, using a fast-start pacing strategy followed by an even pace.

To the best of our knowledge, only one study examined metabolic response during the current XCO race format in an ecological context of simulated cross-country mountain bike racing (Macdermid and Stannard, 2012). This study was conducted with nationally competitive cross-country mountain bike cyclists, and its authors indicated that, during one simulated race-paced lap, the mechanical work produced and the physiological responses were highly variable. This variability is due to the diverse terrain and pacing characteristics, including high-intensity and low-speed pedaling phases during climbing, and high attentional stress during downhill sections presenting technical difficulties. To better define optimal training guidelines for XCO athletes, scientists and coaches need more data on the true requirements of competition with further analysis of the dynamics of the physiological and mechanical responses throughout the race, based on study of several laps and a race time close to that of real competitions.

This study aimed to describe the mechanical and physiological characteristics of this discipline during a full-length simulated race. Specifically, variations in physiological responses and power output were analyzed between laps and uphill or downhill sections. Our initial hypothesis was that modern XCO is a cycling discipline displaying acyclical behavior that leads to specific physiological and mechanical constraints that could differ between laps.

## Materials and Methods

### Participants

The participants of this study were 16 male juniors or U23 mountain bike cyclists all competing at national or international level [Age: 17.4 years (range: 15–23); maximal oxygen consumption: 64.6 ml.min^-1^.kg^-1^ (range: 60.3–72.1); and MAP: 5.2 W.kg^-1^ (range: 4.3–5.6)]. All participants had signed a contract with a team. The contracts include clauses relating to a partnership with the laboratory for collaboration in experiments for data collection (for minors, all contracts were countersigned by parents). The study protocol complied with the standards set out in the Declaration of Helsinki, all participants were volunteers and all experimental procedures were approved by the Local Research Ethics Committee.

### Experimental Design

All the participants took part in two XCO races separated by 1 month. The first race was an official competition that was used as reference for run time. The second was a simulated race organized on the same track as the official race.

One week before performing the simulated race, all participants performed a discontinuous incremental protocol on a cycling-ergometer (Lode Excalibur Sport, Groningen, The Netherlands) to assess their maximal oxygen consumption (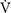
O_2max_), MAP, and ventilatory thresholds. This intermittent incremental protocol has previously been used to determine 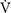
O_2_max for middle-distance runners and to calibrate interval-training protocols (Billat et al., 2000). It has also been used to avoid overestimating athletes who may have high anaerobic capacities (Riboli et al., 2017). Expired gases were measured breath-by-breath by a gas analysis system (K5, Cosmed Srl, Rome, Italy). After a 5 min warm-up at 100 W, the power output was increased by 30 W every 2 min. Each stage was followed by a 30 s rest (Washburn and Seals, 1983). 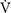
O_2max_ was determined from the four highest 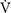
O_2_ values recorded when 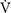
O_2_ reached a plateau at the end of the incremental protocol. The 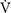
O_2_ plateau was reached when the difference recorded between two consecutive stages was ≤150 ml min^-1^ (Taylor et al., 1955). MAP was defined as the power maintained for more than 1 min at the stage which elicited 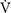
O_2max_ (Billat and Koralsztein, 1996). The first ventilatory threshold (VT1) was defined as the time at which an initial departure from linearity in expiratory volume (VE) was observed, and when a systematic increase in the ventilatory equivalent for O_2_ (VE/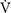
O_2_) and fraction of expired O_2_ (FEO_2_) first appeared. The second ventilatory threshold (VT2) was defined based on a secondary increase in VE and VE/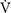
O_2_, and a marked increase in the ventilatory equivalent for CO_2_ (VE/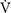
CO_2_) combined with a decrease in the fraction of expired CO_2_ (FECO_2_) (McLellan, 1985). HR was continuously monitored throughout the test using a telemetric HR monitor (Garmin, Switzerland) and the HR sensor of the gas analyzer.

### Simulated XCO Competition

The simulated competition was performed on the same accredited cycling track as the official competition. This track was an outdoor track (length = 5.10 km) with uphill and downhill sections (cumulative altitude difference for the entire trial = 215 m). During the simulated race, after a standardized warm-up, two cyclists of similar performance level (based on national ranking) participated in the same run to mimic competitive conditions. Each run consisted of three laps with a 30 s rest period between laps, during which blood samples were drawn (Gullstrand et al., 1994) and participants allowed to drink *ad libitum*. During these periods, cyclists were allowed to remove the mask from the gas analyzer and breathe freely.

### Physiological and Mechanical Assessment During the Simulated Competition

Gas exchange (K5, Cosmed Srl, Rome, Italy), HR (Garmin, Ltd, Schaffhausen, Switzerland), power output (Rex1 inpower, Rotor, Madrid, Spain) and GPS position (Garmin Edge 520 Ltd., Schaffhausen, Switzerland) were continuously recorded during all runs (recording frequency 1 Hz, except for gas exchange, which was breath-by-breath). To attenuate variability in breath-by-breath 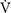
O_2_ related irregular ventilation, and more specifically in this particular exercise due to vibrations affecting ventilation, breath-by-breath and HR data were processed using a moving average over 10 breaths.

Before the start of the race, between each lap and immediately after the race, fingertip blood samples were collected (∼95 μL) from all participants. Capillary blood samples were immediately analyzed for bicarbonate (HCO3^-^), pH (iStat clinical analyzer, Abbott Point of Care, East Windsor, NJ, United States), and blood lactate (Lactate Scout, Senslab, Leipzig, Germany).

Power was measured using a Rotor power meter, which is a single-sided power meter measuring power based on output from strain gauges in the left side crank arm. This systems was recently compared to a number of portable power meters during road cycling (SRM, Powertap, SRAM Quark, Stages powermeter) and showed a high concurrent validity (standard error estimate: 2 W; Bland Altman 95% Limits of Agreement:+ 6W and Intraclass Correlations: 1.00) (Sanders et al., 2017). The power of the Rotor system was calibrated based on the power of the Lode cycling-ergometer. Lap time and overall performance were measured based on GPS values.

### Data Analysis

Oxygen consumption (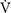
O_2_) and HR values were expressed as %
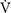
O_2max_ and %HR_max_, respectively, and divided into three physiological intensity zones. The first zone (Z1) was the physiological region below VT1, the second zone (Z2) corresponded to a region between VT1 and VT2, and the third zone (Z3) was located between VT2 and 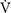
O_2max_.

Power output measured during the different laps was expressed in % of MAP and divided into four power zones (Bernard et al., 2009): P1 below the power corresponding to VT1, P2 between the power corresponding to VT1 and VT2, P3 between the power above VT2 up to MAP, and P4 for the power above MAP. The efforts exerted during P4 were subdivided into five categories based on their duration: 1–5 s, 6–10 s, 11–15 s, 16–20 s, and longer than 20 s. The duration of each time interval, expressed as a percentage of the total time above MAP, and the number of actions for each time interval were reported. Time spent without pedaling was also assessed, it was classed as a fifth power zone named NP.

### Statistical Analysis

All statistical analyses were performed using a statistical software package (STATISTICA for Windows 10; StatSoft, Inc., Tulsa, OK, United States). All data were expressed as mean ± SD. The correlations between the time spent in each zone expressed in %
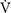
O_2max_ and expressed in %HR and the correlations between the time spent in each zone expressed in %
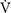
O_2max_ and expressed in %MAP, were calculated using the Pearson correlation coefficient. O_2_ consumption and HR measured prior to the start of the field trial, and the O_2_, HR, and power output data collected during each of the separate laps were placed in their respective physiological intensity and power zones. Repeated measures ANOVA were performed to test differences between each lap, using the amount of time spent in the physiological and power zones as dependent variables. Repeated ANOVA (period) were performed on lactate, pH, and HCO3^-^ values measured before the start of the trial and immediately after each lap. The threshold for statistical significance was set to *p* ≤ 0.05. When an effect was observed, the difference between periods was assessed using a Newman–Keuls *post hoc* test. When a difference was identified, the effect size was calculated based on Cohen’s d (Sullivan and Feinn, 2012). The effect was ranked small (*d* ≥ 0.2), medium (*d* ≥ 0.5), or large (*d* ≥ 0.8).

## Results

### Race Parameters

Whatever the group, no significant difference in performance was found between the simulated race and the official competition (simulated run vs. official race: 64 ± 1.5 min vs. 66 ± 2 min). Climatic conditions were comparable for both races, both in terms of average ambient temperature (17°C vs. 19°C) and relative humidity (61% vs. 54%), and the track was therefore in a similar condition.

### Physiological Demands

The time spent in each VO_2_ zone during the different laps is presented in **Table 1**. Throughout the run, 29.4 ± 10% of the time was spent below VT1, 33.6 ± 2.7% was spent in the second zone, and 37.0 ± 10.9% of the time participants were above VT2. A significant difference between laps was observed in terms of the time spent in Z1 [*F*(2.30) = 17.49, *p* < 0.05] and in Z3 [*F*(2.30) = 11.45, *p* < 0.05]. A moderate increase in time spent in the first zone was recorded between the first and second laps (*d* = 0.74), and between the second and third laps (*d* = 0.46). Overall, a large increase of the time spent in Z1 was observed between the first and the third laps (*d* = 1.16). Simultaneously, a large decrease in time spent in Z3 was observed between the first and third laps (*d* = 1.07). For the time spent in the second zone, differences between laps were not significant [*F*(2.30) = 0.41, *p* = 0.66].

**Table 1:** Percentage of total time spent in each physiological zone for each lap.

		Z1	Z2	Z3
VO_2_ (%_TotalTime_)	Lap 1	19.8 ± 15.6^∗†^	31.4 ± 15.6	48.8 ± 15.1^∗†^
	Lap 2	28.7 ± 16.0^∗‡^	36.6 ± 16.5	34.7 ± 15.6 ^∗‡^
	Lap 3	39.7 ± 13.8^†‡^	32.9 ± 17.3	27.4 ± 16.0^†‡^
HR (%_TotalTime_)	Lap 1	17.1 ± 8.9	53.2 ± 8.6	29.7 ± 6.2
	Lap 2	12.9 ± 11.1	52.5 ± 9.3	34.6 ± 5.7
	Lap 3	15.4 ± 8.4	50.3 ± 12.1	34.2 ± 9.3


*Data are presented as mean ± standard deviation, with results of ANOVA comparisons (^∗^significance difference between Lap1 and Lap2; ^†^significance difference between Lap1 and Lap3; ^‡^significance difference between Lap2 and Lap3).*

### Heart Rate

The time spent in each HR zone for the different laps is presented in **Table 1**. Over the whole run, 15.1 ± 2.1% of the time was spent at a HR corresponding to the zone below VT1, 52.1 ± 1.5% of the time was spent in the second zone, and 32.8 ± 2.7% of the time was spent at a HR above that recorded for VT2. No significant difference between laps was observed for the different zones [Z1: *F*(2.30) = 1.13, *p* = 0.34; Z2: *F*(2.30) = 0.03, *p* = 0.96; and Z3: *F*(2.30) = 1.02, *p* = 0.38].

### Power Output

The time spent in each power output zone for the different laps is presented in **Table 2**. Throughout the run, participants spent 19.2 ± 5.0% of the time at NP, 30.6% ± 9.4% at P1, 12.6 ± 5.0% at P2, 9.4 ± 4.6% at P3 and 28.2 ± 8.1% at P4. A significant difference between laps was observed only for time spent in the P4 zone [*F*(2.30) = 3.67, *p* = 0.03], with a measureable decrease between the first and second laps (*d* = 0.62), and a smaller decrease between the second and third lap (*d* = 0.41). As a result, a large effect was found between the first and third laps (*d* = 1.00). For the time spent in the other zones, differences between laps were not significant [NP: *F*(2.30) = 0.16, *p* = 0.85; P1: *F*(2.30) = 1.61, *p* = 0.21; P2: *F*(2.30) = 0.30, *p* = 0.74; P3: *F*(2.30) = 0.09, *p* = 0.92]. However, it should be noted that the average time spent in P1 increased with each lap, and concomitantly, the time spent in P4 decreased; times for NP, P2, and P3 remained stable across all laps.

**Table 2:** Percentage of total time spent in each power output zone for each lap.

		NP	P1	P2	P3	P4
Power (%_TotalTime_)	Lap 1	18.8 ± 4.3	27.0 ± 8.1	11.9 ± 4.9	9.5 ± 5.1	32.8 ± 8.2^∗†^
	Lap 2	18.9 ± 4.6	31.2 ± 9.8	12.3 ± 5.2	9.7 ± 4.3	27.9 ± 7.9^∗‡^
	Lap 3	19.8 ± 6.0	33.5 ± 10.2	13.6 ± 5.0	9.1 ± 4.5	24.0 ± 8.2^†‡^


*Data are presented as mean ± standard deviation, with results of ANOVA comparisons (^∗^: signif. difference between Lap1 and Lap2; ^†^significance difference between Lap1 and Lap3; ^‡^significance difference between Lap2 and Lap3).*

**FIGURE 1:**
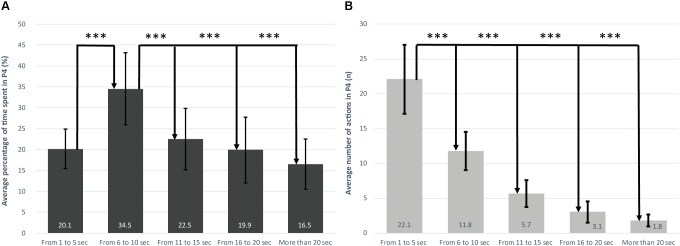
**(A)** Percentage of time spent in P4 by 5 s segments. **(B)** Number of actions in P4 by 5 s segments. When a difference between laps was significant, Cohen’s d was calculated (^∗^*d* ≥ 0.2, ^∗∗^*d* ≥ 0.5, ^∗∗∗^*d* ≥ 0.8).

For the P4 zone, a significant effect was observed on the percentage of total time spent in the different 5 s durations over which the effort exerted exceeded MAP [*F*(4.180) = 24.24, *p* < 0.05]. The time spent above MAP most frequently lasted 5–10 s as compared to other maintenance durations (i.e., 1–5 s, 11–15 s, 16–20 s and more than 20 s; *d* = 1.28; *d* = 1.23, *d* = 1.57, *d* = 2.07, respectively) (**Figure 1A**). A significant effect was also observed for the number of actions performed in P4 [*F*(4.180) = 24.24, *p* < 0.05]. The number of efforts in the 1–5 s time category (*n* = 22.1, representing almost 50% of efforts in P4) was significantly higher than each of the other four categories (*d* = 2.48, *d* = 3.71, *d* = 4.91, *d* = 5.51, respectively) (**Figure 1B**).

### Blood Analysis

**FIGURE 2:**
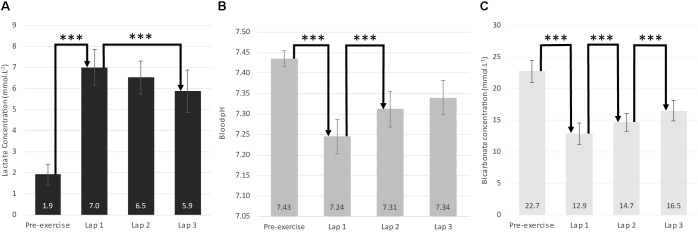
Pre-exercise and post-lap blood analyses. **(A)** Lactate concentration. **(B)** Blood pH. **(C)** Bicarbonate concentration. When a difference between laps was significant, Cohen’s d was calculated (^∗^*d* ≥ 0.2, ^∗∗^*d* ≥ 0.5, ^∗∗∗^*d* ≥ 0.8).

Mean lactate concentration in pre-exercise was 1.9 ± 0.5 mmol^-1^ and over the whole run the mean lactate value was 6.5 ± 0.9 mmol^-1^. A significant difference was observed in lactate values between laps [*F*(3.45) = 85.41, *p* < 0.05], with a decrease between the end of the first lap and the end of the third lap (*d* = 1.21) (**Figure 2A**).

Mean pre-exercise pH and HCO3^-^ were 7.4 ± 0.0 and 22.7 ± 1.7 mmol^-1^, respectively. Over the whole run, the mean pH and HCO3^-^ were 7.3 ± 0.0 and 14.7 ± 1.6 mmol^-1^, respectively. A significant effect of period was observed for blood pH and HCO3^-^ [*F*(3.45) = 66.1, *p* < 0.05; *F*(3.45) = 69.32, *p* < 0.05, respectively], with a large and significant decrease between pre-exercise levels and levels measured at the end of the first lap (pH: *d* = 5.79; HCO3^-^: *d* = 5.76). An increase was also observed between the end of the first lap and the end of the second lap (pH: *d* = 1.55; HCO3^-^: *d* = 1.15). In addition, for HCO3^-^ a further increase was observed between the second and third laps (*d* = 1.19) (**Figures 2B,C**).

Correlations between physiological and mechanical analysis of the XCO race.

**FIGURE 3:**
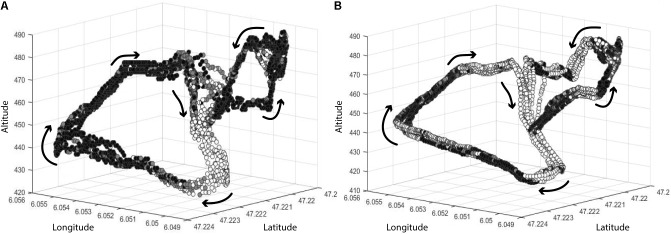
**(A)** VO_2_ zones as a function of GPS position. **(B)** Power zones as a function of GPS position. (White dots correspond to Z1 or NP+P1; gray dots correspond to Z2 or P2; black dots correspond to Z3 or P3+P4).

No significant correlations were found between the time spent in intensity zones expressed as %
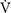
O_2max_ and intensity zones expressed either as %HR_max_ (*r* = -0.01) or %MAP (*r* = -0.02). To determine the relative interest of each analysis, the evolution of 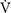
O_2_ and Power were represented as a function of GPS position and intensity zones (**Figures 3A,B**, respectively).

## Discussion

The aim of this study was to describe the physiological and mechanical characteristics of the current mountain bike XCO race and to analyze the dynamics of these parameters between laps. The main findings were as follows:

- During a mountain biking XCO race, a significant part of the race is performed above the second ventilatory threshold or even beyond MAP, indicating a high level of solicitation of both aerobic and anaerobic metabolic pathways; furthermore, the respective contributions of these pathways vary between laps.

- Periods spent beyond MAP tend to be short bursts, oscillating mainly between 5 and 10 s, suggesting that mountain biking has a similar acyclical profile to intermittent team sports such as soccer or basketball (Reilly et al., 2000; Cormery et al., 2007).

- Classical field parameters such as %HR or %MAP do not correlate with the physiological demands expressed as %
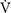
O_2max_, which might thus be the best parameter to monitor the physiological demands of mountain bike XCO.

Classically, the physiological demands of cycling events in field studies are assessed based on HR recordings (Lucía et al., 1999; Palmer et al., 1999; Padilla et al., 2000; Impellizzeri et al., 2002) and/or power output (Hansen et al., 1999; Smith et al., 2001; Stapelfeldt et al., 2004; Macdermid and Stannard, 2012), since power output is a direct indicator of mechanical performance (Coyle et al., 1991). The HR values recorded in this study were very similar to those recorded in previous studies analyzing the old XCO format (Stapelfeldt et al., 2004; Impellizzeri and Marcora, 2007). For example, Impellizzeri et al. (2002) reported that 18% of the time was spent in a HR zone under VT1, 52% was spent between VT1 and VT2, and 30% was spent above VT2, a split which is very close to our findings (**Table 1**). Thus, based on HR data, our results indicated that the current XCO format is not significantly different from the old one. However, the distribution of power output values recorded in our study were slightly different from those presented in previous studies, one possible explanation could be related to the fact that the XCO race duration was shortened in 2007. For example, Stapelfeldt et al. (2004) reported that 39 ± 6% of race time was spent in P1, 19 ± 6% was spent in P2, 20 ± 3% of race time was spent in P3, and 22 ± 6% was spent in P4. The increased time spent in P1 observed in the modern format could be related to the need to recover between the explosive actions performed in P4. This observation highlights the altered dynamics of the effort in modern format, characterized by shorter durations and more technical sections.

A novelty of this study was that we measured 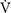
O_2_ throughout the race and between laps. This monitoring approach revealed that the time spent in different physiological zones based on 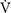
O_2_ values did not correlate with either HR or power output. This difference could be related to the fact that 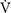
O_2_ data reflect high-intensity bouts and the recovery periods between these bouts, whereas power reflects power output recorded in the crank arm. In XCO races, technical parts of the race require driving that involves significant upper limb solicitation. This effort is not represented in measurements of power. This result reveals the difficulty in technical sports of relating power output to physiological demands, and thus of describing activity constraints only based on mechanical data provided by cycling parts. In **Figure 3**, which shows a 3D representation of both 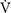
O_2_ and power data, in the upper parts of the track, specifically at the end of the climbs and at the beginning of the descending portions, it can be noted that the 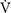
O_2_ remains in high-intensity domains. This result contrasts with the power measurements. During these recovery periods, power data provide little or no information. The lack of correlation between HR and 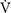
O_2_ has previously been reported in intermittent sports (Bangsbo et al., 2007). In the XCO context, the use of HR values is even more limited due to the presence of static, eccentric and concentric phases (Dean, 1988; Arimoto et al., 2005). Moreover, our participants were allowed to drink only between laps during the 30 s rest, thus they may have become dehydrated during the race, a condition which would affect HR drift and increase the difference between 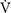
O_2_ and HR data (Coyle, 1998). Finally, HR could also be affected by the mental load (Blitz et al., 1970), which is particularly prominent during the technical parts of the race and when the risk of falling is high. When expressed in %
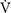
O_2max_, our values indicate that both anaerobic and aerobic contributions are solicited during an XCO race, with more than 70% of race time spent above VT1, and a mean intensity of 87% 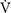
O_2max_. Moreover, an increase in blood lactate (to 6.5 mmol.l^-1^) and a decrease in pH values (to 7.29) reflected the extent to which anaerobic metabolism was solicited. Our results also clearly demonstrate the intermittent nature of the XCO race format, with significant use of very short-lived efforts, and a significant proportion of time spent delivering little or very low power (NP). This race dynamic was illustrated in the P4 period by the large number of actions performed during the 1–5 s duration zone (*n* = 22.1 on average) and by the time spent in the 5–10 s period (34.5 ± 8.6% P4 total time), suggesting that most of the actions performed in P4 last less than 10 s. These phases often involve static and eccentric contractions of upper body muscles and are associated with an attentional workload that could accelerate the onset of fatigue (Mehta and Agnew, 2012). For example, during technical downhill phases (with a steep slope and frequent roots or rocks), athletes do not pedal, but their muscles are solicited for shock absorption and to maintain balance.

Another novel approach used in the present study was the analysis and comparison of data from each individual lap of the simulated race. Recent studies indicated that during the current XCO format cyclists adopt a fast-starting pacing strategy followed by positive pacing (Granier et al., 2018; Viana et al., 2018). Metabolic data recorded in our study corroborate these observations, since during the first lap a larger proportion of the time was spent at high intensity levels (P4 and Z3) compared to during laps 2 and 3. The first lap was also associated with a significant decrease in blood pH and HCO3^-^ and a significant increase in blood lactate compared to pre-exercise levels. Blood lactate, pH, and HCO3^-^ tended to return toward pre-exercise levels following laps 2 and 3, but still remained significantly different to pre-exercise values. These observations reflect high solicitation of the anaerobic pathway in the first lap and a gradual return to a more aerobic exercise regimen during subsequent laps. This result could be explained by the mass-start in the XCO race format, as described by some previous studies (Impellizzeri and Marcora, 2007; Macdermid and Stannard, 2012), which requires athletes to position themselves ahead of the race to avoid being slowed or hindered by other cyclists when they find themselves on the narrow path or in technical sections of the track. Due to technical constraints, in our study, each run was conducted with only two cyclists on the track at the same time to mimic competitive conditions and the motivational environment of a race, but our results nevertheless agree with pacing analysis reported during a real race (Granier et al., 2018).

The results reported in this study have some practical applications for coaches, athletes or scientists who wish to develop and optimize training programs. For example, a weekly training program should combine high- and low-intensity aerobic sessions, as well as including sessions to improve repeat sprint ability (RSA) and high-intensity intermittent endurance. Bishop et al. (2011), in their narrative review of RSA training, recommend two training approaches: (i) specific training to perform repeated sprints, and resistance training; (ii) working on the limiting factors for RSA (metabolic factors such as oxidative capacity, recovery, and H^+^ buffering; and neural factors like muscle activation and recruitment strategies). For XCO cyclists, a typical training week could include 2–3 high-intensity intermittent training sessions incorporating numerous jumps, climbs and descents to mimic the demands of the race. Due to the high solicitation of the upper limbs during these technical portions of the race, specific work should be included to reinforce these muscle groups. For this type of exercise, mountain riders can focus on resistance training. These practical recommendations are supported by the results reported by Edge et al. (2006) or Hill-Haas et al. (2007), indicating significant improvements in RSA performance after a training program composed of six leg exercise for 2–5 sets at 15–20 RM 3 days per week over 5 weeks. Thus, specific muscle building activity while maintaining a favorable power-weight ratio could be recommended for XCO athletes.

## Conclusion

Our results indicate that the current XCO format represents an acyclical and intermittent sport which differs from classical cycling events and is closer to some high-intensity team sports. The relevance of 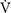
O_2_ values, compared to HR and power output values, when monitoring XCO performance is highlighted. Some results from this study may have direct implications for training strategies, such as the identification of typical bursts above MAP and the potential lack of relevance of powermeters to monitor performance. Further studies will be needed to better understand the metabolic mechanisms and muscular fatigue associated with this sport, as well as the extent of muscle damage induced by eccentric exercise, or the impact of mental workload on XCO performance. Finally, the results from this study emphasize the need for relevant training strategies to optimize performance and recovery.

## Author Contributions

AH is the lead author, designed the study, performed the experiments, processed the data, and wrote the paper. SD performed the experiments and processed the data. DB and L-aM performed the experiments. JB designed the study, processed the data, and wrote the paper.

## Conflict of Interest Statement

The authors declare that the research was conducted in the absence of any commercial or financial relationships that could be construed as a potential conflict of interest.
